# Repeated remote ischemic preconditioning and isoflurane anesthesia in an experimental model of renal ischemia-reperfusion injury

**DOI:** 10.1186/s12871-017-0310-x

**Published:** 2017-01-28

**Authors:** Theo P. Menting, Mehmet Ergun, Moira H. D. Bruintjes, Kimberley E. Wever, Roger M. L. M. Lomme, Harry van Goor, Michiel C. Warlé

**Affiliations:** 1 Department of Surgery, Radboud University Medical Center, Geert Grooteplein-zuid 10, 6525 GA Nijmegen, The Netherlands; 2 Systematic Review Centre for Laboratory animal Experimentation, SYRCLE, Radboud University Medical Center, Geert Grooteplein-zuid 10, 6525 GA Nijmegen, The Netherlands

**Keywords:** Anesthetic preconditioning, Animal experiment, Ischemia reperfusion injury, Kidney, Repeated remote ischemic preconditioning

## Abstract

**Background:**

In animal studies, remote ischemic preconditioning (RIPC) and anesthetic preconditioning are successful in reducing renal ischemia reperfusion injury (IRI), however the protective effect of RIPC may be improved by repeating the RIPC stimulus.

**Methods:**

Sprague-Dawley rats underwent unilateral nephrectomy followed by 30 min of renal pedicle clamping. Animals were allocated into six groups: sham, control (IRI), RepISO (daily isoflurane anesthesia), RIPC (single dose isoflurane anesthesia and single dose RIPC), RepISO + RIPC (7-day isoflurane anesthesia and single dose RIPC) and RepISO + RepRIPC (7-day isoflurane anesthesia with 7-day RIPC). RIPC was applied by 3×5 min of cuff inflation on both thighs. Serum creatinine and urea levels were measured and histology was obtained at day two.

**Results:**

RepISO diminished renal IRI, as reflected by a significant reduction in serum creatinine levels as compared to the control group, 170 ± 74 resp. 107 ± 29 μmol/L. The other preconditioning protocols showed similar reduction in serum creatinine levels as compared to the control group. No significant differences were observed between the different preconditioning protocols. For urea levels, only RepISO + RIPC resulted in significantly lower levels as compared to the control group, 14 ± 4 resp. 22 ± 7 mmol/L (*p* = 0.010). In the preconditioning groups only RepISO showed less histological damage as compared to controls 1.73 ± 1.19 resp. 2.91 ± 1.22 (*p* = 0.032).

**Conclusions:**

In this study no additional protective effect of repeated ischemic preconditioning was observed as compared to single dose RIPC. Repeated administration of isoflurane provided stronger protection against renal IRI as compared to single dose isoflurane.

## Background

Ischemia reperfusion injury (IRI) is tissue damage caused by the restoration of blood flow after a period of deprived circulation of that tissue [1]. The deficit of oxygen and nutrients during the ischemic phase creates a condition in which the return of blood flow induces oxidative stress, inflammation and results in apoptosis of the cell [2]. This may lead to tissue damage and loss of organ function [3]. The kidney is an organ especially vulnerable to IRI, due to its high-energy demand and delicate microcirculation. IRI of the kidney is a significant clinical problem in shock, renal transplantation and major cardiac or vascular surgery [4]. A promising method to diminish IRI was first described in 1986 by Murry [5], he discovered that short harmless periods of ischemia can protect the heart against a prolonged ischemic period; this phenomenon is called ischemic preconditioning (IPC). It was later described that the interruption of blood flow to an organ different than the target organ could also have a protective effect on IRI. This phenomenon is known as remote ischemic preconditioning (RIPC) [6]. Although the exact mechanism of RIPC is unknown, prevention of apoptosis by closure of the mitochondrial permeability transition pores (mPTP), seems to play a pivotal role [3]. A limb is often used as the remote organ for the application of the RIPC stimulus as the blood flow can safely and easily be obstructed by insufflation of a blood pressure cuff around an arm or leg. Experimental studies have shown that RIPC does not only protect against IRI in the heart, but also in other organs, including the kidney [7].

Not only a distant ischemic impulse can cause renal protection from IRI, some anesthetics also protect the kidney against IRI. In myocardial and renal animal studies, [8] anesthetics have shown to reduce IRI in a similar signaling cascade as RIPC, known as anesthetic preconditioning (APC). Volatile anesthetics have extensively been tested for their APC effectiveness in cardiac studies: isoflurane, sevoflurane, desflurane [9, 10], halothane [11] and ether derived anesthetics [12] have proven clinical and preclinical cardioprotective effects. Experiments with intravenous anesthetics, propofol, barbitarates and ketamine [13–15] show no protective effect and have been demonstrated to inhibit mKATP channels which is an indication these anesthetics might diminish the protective effect of APC or RIPC [16]. The effects of multiple periods of anesthetics on IRI are unknown.

In general, animal studies show that RIPC is effective in reducing renal IRI [17]; however, human studies show disappointing results, with a small or non-significant protective effect [18, 19]. Cumulating evidence exists that in cardiac IRI models, repeating the RIPC stimulus over a period of multiple days, repeated RIPC (RepRIPC), could be more effective as compared to single dose RIPC [13, 20, 21]. It is unclear if this holds true for renal IRI. In this study we test whether the null-hypothesis could be rejected that single dose and repeated RIPC are equally effective in an experimental model of renal IRI.

## Methods

The Committee for Animal Experiments of the Radboud Medical Center, Nijmegen approved all procedures (registration number 20149), and the experiment was conducted according to the ARRIVE criteria. 59 male Sprague–Dawley rats (Harlan Laboratories, Eystrup, Germany) were brought into the facility two weeks before the start of the experiment to acclimatize. Rats from different groups were housed randomly in the same room and under standard specific pathogen-free housing conditions. The environmental temperature was regulated at 22 °C, with a relative humidity of 45% and a 12/12h day/night cycle. At the start of the experiment the animals weight was 311 ± 21g, at the age of 10 weeks.

### Blinding

Group assignment of each rat was done by computer-generated randomization. The surgeon, caregivers and the analysts performing creatinine, urea and histology measurements were blinded for group assignment of the animals.

### Study design

**Fig. 1:**
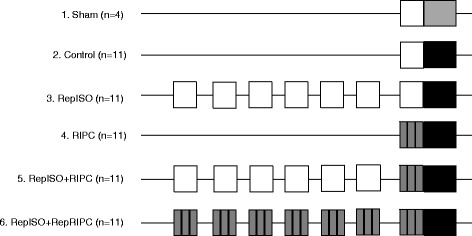
Schematic protocol of the animal groups were the line is a non linear timeframe of seven days. The open boxes indicate a period of anesthesia alone, gray boxes a period or RIPC and black boxes a period of renal ischemia. Animals were randomly allocated into six groups: sham, control (IRI), RepISO (daily isoflurane anesthesia), RIPC (single dose isoflurane anesthesia and single dose RIPC), RepISO + RIPC (7-day isoflurane anesthesia and single dose RIPC) and RepISO + RepRIPC (7-day isoflurane anesthesia with 7-day RIPC). RIPC was applied by 3×5 min of cuff inflation on both thighs

All animals were anesthetized using isoflurane for the same period of time and all animals underwent right nephrectomy. Animals were randomly divided in six groups (Fig. 1): Group 1 and 2 underwent no preconditioning. The sham group (*n* = 4, group 1) underwent a laparotomy, including the resection of the right kidney. The control group (*n* = 11, group 2) underwent 30 min of left renal ischemia (IRI stimulus) during right kidney resection. Groups 3–6 were the experimental, preconditioning groups, all undergoing 30 min of left renal ischemia at the day of surgery and a specific preconditioning stimulus: In group 3; repeated isoflurane (RepISO, *n* = 11), the animals underwent seven days of isoflurane anesthesia for 25 min prior to the day of the operation. In group 4; single RIPC (RIPC, *n* = 11); the animals underwent 3× 5 min of cuff inflation and 5 min of reperfusion prior to the operation. Cuff inflation was initiated by using human toe pressure cuffs, inflating them simultaneously to 200mmHg on both thighs. RIPC required 25 min of anesthesia as the last 5 min of reperfusion did not require anesthesia. In group 5; repeated isoflurane and a single RIPC stimulus (RepISO + RIPC, *n* = 11), the animals underwent seven days of anesthesia for 25 min prior to the day of the operation. On the day of surgery, during isoflurane anesthesia, 3× 5 min of cuff inflation on both thighs and 5 min of reperfusion was performed. In group 6; repeated isoflurane and repeated RIPC (RepISO + RepRIPC, *n* = 11), the animals underwent seven days of anesthesia for 25 min together with seven days of 3× 5 min of cuff inflation on both thighs and 5 min of reperfusion.

### Surgical procedures

All experiments were randomly performed between 8.00 and 16.00h on Mondays and Tuesdays. Preoperative analgesic [Carprofen, 5mg/kg body weight (b.w.)] was administered subcutaneously 30 min prior to surgery. Surgical procedures were conducted using standard aseptic surgical techniques and all microsurgical instruments were sterilized using a dry bead sterilizer (Inothech, Dottikon, Switzerland). Animals were placed on a sterile drape overlying a heating pad to maintain body temperature at 36–38 °C, monitored continuously using a rectal thermometer. Body weights were recorded prior to surgery, prior to blood collection and at the end of the experiment. Anesthesia was induced with 5% isoflurane in pressurized air and maintained at 2.5–3%. Depth of anesthesia was assessed by toe and tail pinch. Preconditioning was done by TM. All operations were done by an experienced microsurgeon (RL), renal ischemia was initiated by blunt dissection of the left renal hilus, and an atraumatic vascular clamp was used to obstruct the venous and arterial blood flow of the kidney. Complete obstruction was confirmed by visualization of the kidney gaining the typical ischemic dark purple color; complete revascularization after removal of the clamp was also visualized before closure of the abdomen. Closure of the abdomen was done by a running suture, securing both ends with a metal clip to prevent opening of the wound by the animal. One day post-operatively, an analgesic (Carprofen, 5 mg/kg b.w.) in 5mL saline was administered subcutaneously.

### Renal function analysis and histology

At baseline, day one and day two blood samples were collected and stored. Blood samples were collected in EDTA tubes and centrifuged for 15 min at 3000g to obtain plasma. Plasma was snap frozen in liquid nitrogen and stored at -80 °C until further use. For the histology, tissue from the remaining kidney was taken two days after surgery and was fixed in 4% paraformaldehyde for at least 48h. For light microscopy of the renal cortex, kidneys were dehydrated and embedded in paraffin. To score renal damage, sections of 4 μm were stained with periodic acid-Schiff. Of each kidney, four sections were taken at different latitudes and scored for damage of the renal cortex and averaged. Damage scoring was performed by a blinded investigator, on a scale from 0 to 4 according to the Jablonski scale [22], with 0: no proximal tubule damaged, and 4: all tubules damaged.

### Statistical and power analysis

Serum creatinine levels were used as the primary outcome measure. Previous experiments have shown that in our model of 30 min renal injury, serum creatinine levels in control animals 48h post-operative are on average 290μmol/l, with an average standard deviation of 103μmol/l [23, 24]. We aim to detect a difference in serum creatinine between the RepISO + RepRIPC and all the other experimental groups including the control group of 100μmol/l. Since there are five comparisons we have adjusted our level of significance for five comparisons, using Bonferonni correction: 0.05/5 = 0.01. In order to achieve a statistical power of at least 80%, we require 11 animals per group. Previous experiments have shown that the standard deviation in sham-operated animals is low (average serum creatinine 48h post-operative = 46 ± 8). Therefore 4 animals in the sham group were required. Although the animals were obtained from a different supplier, we estimated that the susceptibility to renal IRI would be similar because the strain, age, sex and weight were identical as in the previously mentioned experiments. All data are presented as mean ± SD unless otherwise specified. The means of the different groups were compared using the Student-t test. The level of statistical significance was set at *p* <0.05. Data were assessed and SPSS 22 and GraphPath 5.03 plotted graphs.

## Results

### Peri-operative complications

Fifty-nine rats were randomly assigned to six different groups. Two rats died during anesthesia. A third animal was excluded at day two of the experiment due to intestinal rotation with obstruction. A fourth rat was excluded because the remaining kidney contained a large tumor, which filled one third of the kidney’s volume. The excluded rats belonged to different groups: control, RepISO, RIPC and RepISO + RepRIPC. The weight of the animals at baseline and the average weight loss at day two in the different groups were not significantly different between the groups.

### Renal function analysis

**Fig. 2:**
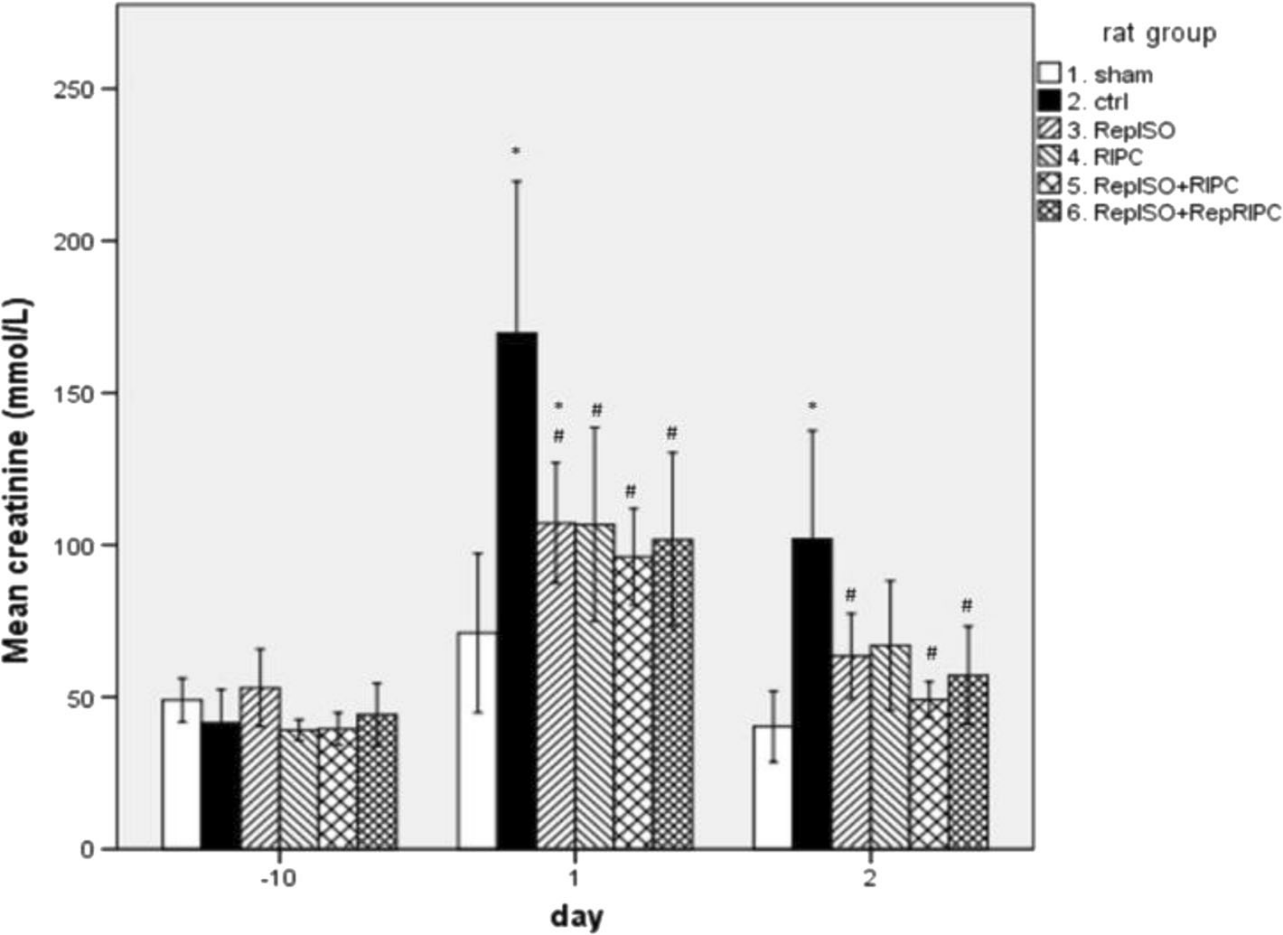
Serum creatinine; day -10 (baseline), 1 and 2 postoperative (* significantly different from sham, # significantly different from control group)

**Fig. 3:**
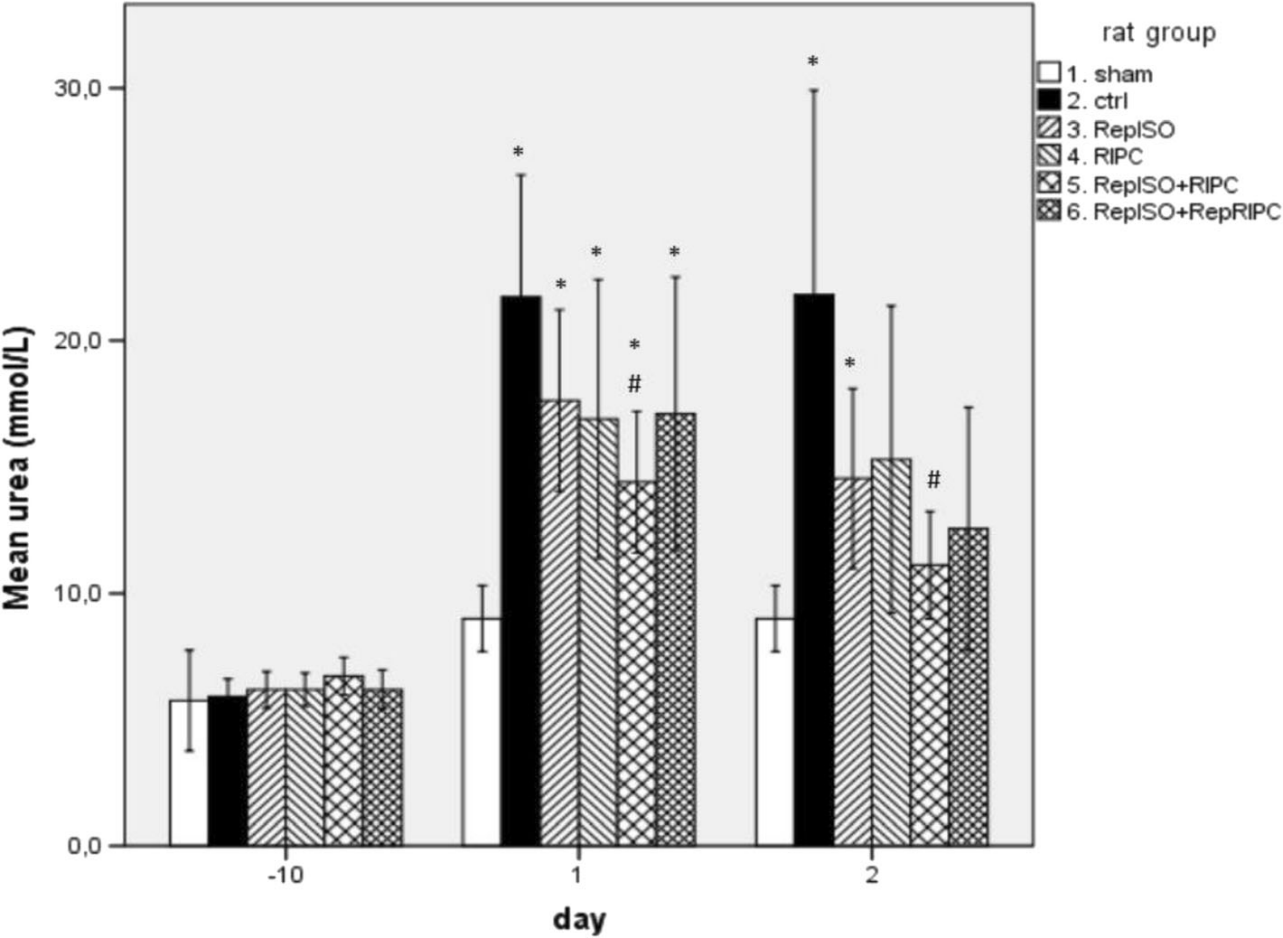
Serum urea; day -10 (baseline), 1 and 2 postoperative (* significantly different from sham, # significantly different from control group)

Serum creatinine (Fig. 2) and serum urea concentrations (Fig. 3) were measured at baseline (ten days before surgery) and on postoperative day one and two. All baseline outcome measures were not significantly different.

In comparison with the control group, all groups showed a significantly lower level of creatinine; control 170 ± 74 μmol/L vs. sham and experimental group 1 and 3–6 respectively; 71 ± 16 μmol/L (*p* = 0.023), 107 ± 29 μmol/L (*p* = 0.022), 107 ± 45 μmol/L (*p* = 0.032), 96 ± 22 μmol/L (*p* = 0.007) and 102 ± 37 μmol/L (*p* = 0.023). For the experimental groups only serum creatinine levels of RepISO on day 1 were significantly higher than sham creatinine levels; 107 ± 29 μmol/L resp. 71 ± 16 μmol/L (*p* = 0.039).

On day two the creatinine concentrations were reduced compared with day one and on day two there was no significant difference between sham and the experimental groups. The control animals showed significantly higher creatinine concentrations compared with the experimental groups, RepISO, RepISO + RIPC and RepISO + RepRIPC respectively: 102 ± 29 μmol/L vs. 63 ± 21 μmol/L (*p* = 0.036), 49 ± 8 μmol/L (*p* = 0.006) and 57 ± 21 μmol/L (*p* = 0.028).

For urea levels, all groups showed significantly higher levels on day one as compared to sham: 9 ± 1 mmol/L vs. control 22 ± 7 mmol/L (*p* = 0.000), vs. RepISO 18 ± 5 mmol/L (*p* = 0.000), vs. RIPC 17 ± 8 mmol/L (*p* = 0.010), vs. RepISO + RIPC 14 ± 4 mmol/L (*p* = 0.002) and vs. RepISO + RepRIPC 17 ± 7 mmol/L (*p* = 0.047). Compared to control operated animals, only serum urea levels in RepISO + RIPC were significantly lower, 14 ± 4 vs. 22 ± 7 mmol/L (*p* = 0.010).

### Histology

**Fig. 4:**
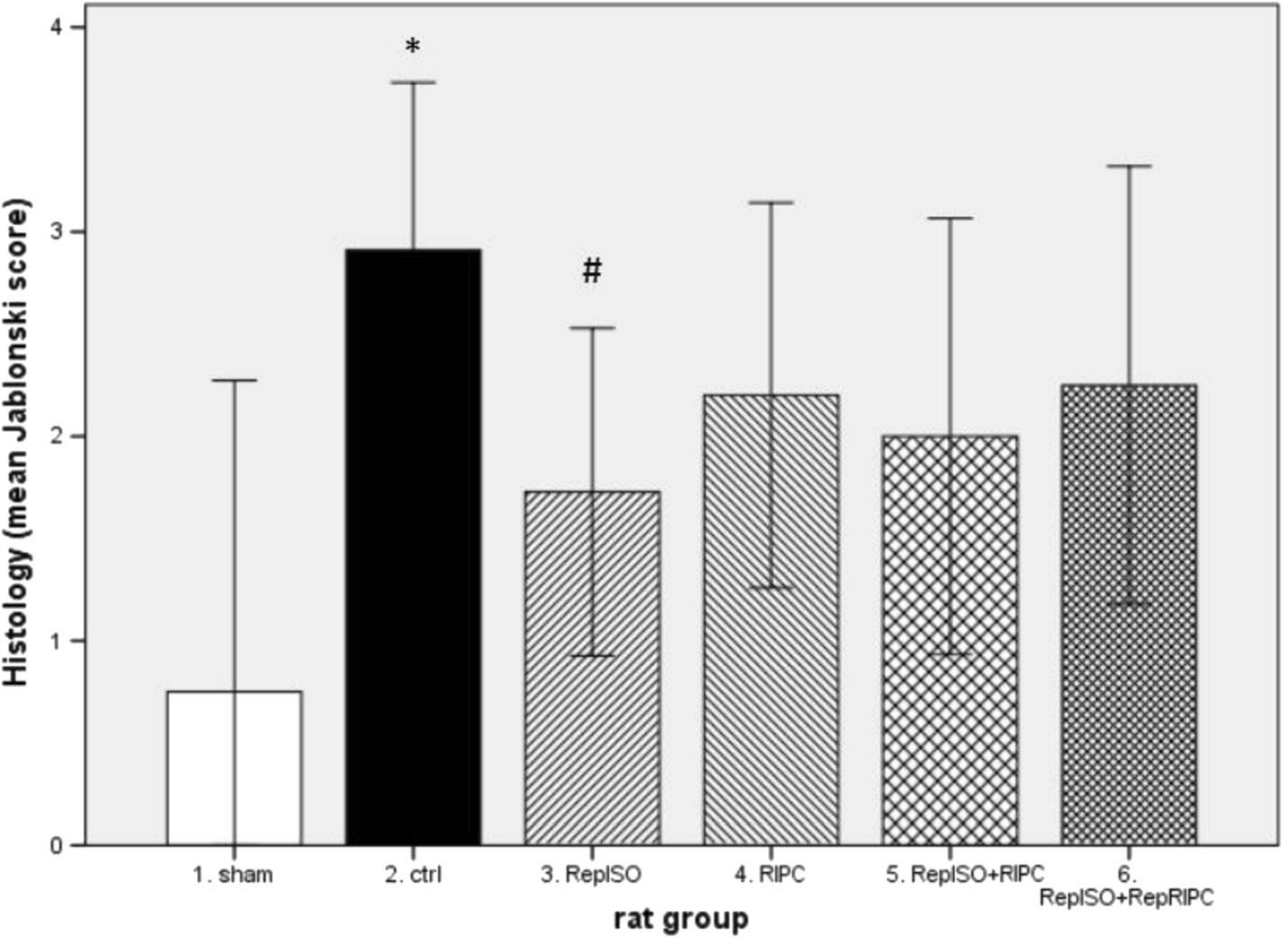
Histology; day 2 postoperative (* significantly different from sham, # significantly different from control group)

Histology, according to the Jablonski score, showed significantly more renal damage in the control group 2.91 ± 1.22 as compared to sham 0.75 ± 0.96 (*p* = 0.007). In the preconditioning groups only RepISO, 1.73 ± 1.19 (*p* = 0.032), showed significantly less damage as compared to control (Fig. 4).

## Discussion

To our knowledge this is the first experiment of RepRIPC compared to single dose RIPC in an experimental renal IRI model. With regard to the primary hypothesis, we were not able to demonstrate an additive protective effect of a repeated ischemic preconditioning stimulus in this experiment. However the question whether an additive protective effect of RepRIPC does not exist or the unanticipated large reduction in renal IRI by repeated isoflurane blurred the additional protective effects of repeated RIPC, remains unanswered.

Results show that all different preconditioning protocols, including RepRIPC, showed a significant reduction in serum creatinine at day one, which was the primary outcome measure. However it is important to note that the observed differences in serum creatinine levels at day one between the different preconditioning protocols and the control group (single dose APC) were smaller than the difference used for the power calculation (100 μmol/L). This indicates that a smaller difference in serum creatinine levels would have been more appropriate to reduce the risk of a type I error. With regard to serum urea levels, only RepISO + RepRIPC showed a significant reduction as compared to the control group, receiving a single period of isoflurane. Probably the number of animals per group was too small to detect differences in serum urea levels between RepISO and controls. With regard to the histology data, only animals in the RepISO group had lower scores for renal injury as compared to controls. This finding supports the main observation of this study, repeated administration of isolfurane provides stronger protection against renal IRI as compared to single dose isolfurane.

In this study isoflurane was chosen as an anesthetic because it is safe, has little side effects and is widely used in animal studies and in patients. The downside of using isoflurane in this experiment is the protective effect of isoflurane on renal IRI. One previous study [8] showed that single dose isoflurane preconditioning ameliorated IRI of the kidney. In our study we showed that a 7-day repeated isoflurane preconditioning provided significantly more protection against renal IRI as compared to single dose isoflurane in the control group. The smallest number of daily repeated isoflurane preconditioning cycles providing maximum protection remains unknown. To our knowledge, the strong protective effects of repeated isoflurane administrations over multiple days has not been described previously.

Another remarkable observation is that 30 min of pedicle clamping induced less renal injury as compared to our previous experiments [23, 24]. As the amount of renal injury varied between this experiment and previous observations, it would have been better to include more animals to control for this variation in our experimental model. The most likely explanation for the difference with the previous experiments is that our animals were obtained from a different supplier. Despite the fact that we used the same strain, there may have been differences in the genetic makeup leading to a lower susceptibility to renal IRI. This phenomenon is supported by studies showing that different strains of mice have a different susceptibility to cardiac IRI [25, 26]. In this experiment 30 min of IRI was chosen, despite the fact that 45 min is more commonly used in similar experiments [17]. The reason to shorten the IRI period is that the amount of IRI in most animal studies is relatively large as compared to clinical trials [17–19, 21, 27]. In our view the induction of a lower amount of IRI results in a more realistic animal model for the translation into clinical practice.

## Conclusion

IPC has been a promising phenomenon since its discovery in 1986 [5]; however, the vast amount of IRI protection by IPC, shown in animal studies, cannot be translated into clinical trials [17, 19]. Accumulating evidence indicate that RepRIPC is a promising tool to provide a more effective and robust RIPC stimulus. RepRIPC was successfully studied in animal heart models [28, 29], endothelial dysfunction models in healthy humans [21] coronary artery bypass grafting [30] and after stroke [20]. Nevertheless our results show that it is difficult to establish additional protection of a repeated RIPC stimulus as compared to single dose RIPC in animal studies reducing renal IRI. In future animal studies investigating the mechanisms and/or efficacy of repeated RIPC and APC, the strong protective effects of the repeated administration of (volatile) anesthetics, i.e. isoflurane, should be taken into account.

## Abbreviations

APC: Anesthetic preconditioning
IPC: Ischemic preconditioning
IRI: Ischemia reperfusion injury
mPTP: Mitochondrial permeability transition pores
RepISO: Repeated isoflurane anesthesia
RepRIPC: Repeated remote ischemic preconditioning
RIPC: Remote ischemic preconditioning

## References

[1] Bonventre JV (1988). Mediators of ischemic renal injury. Annu Rev Med.

[2] Piper HM, Garcia-Dorado D, Ovize M (1998). A fresh look at reperfusion injury. Cardiovasc Res.

[3] Ong SB (2015). The mitochondrial permeability transition pore and its role in myocardial ischemia reperfusion injury. J Mol Cell Cardiol.

[4] Schrier RW, Wang W (2004). Acute renal failure and sepsis. N Engl J Med.

[5] Murry CE, Jennings RB, Reimer KA (1986). Preconditioning with ischemia: a delay of lethal cell injury in ischemic myocardium. Circulation.

[6] Przyklenk K (1993). Regional ischemic ‘preconditioning’ protects remote virgin myocardium from subsequent sustained coronary occlusion. Circulation.

[7] Cochrane J (1999). Ischemic preconditioning attenuates functional, metabolic, and morphologic injury from ischemic acute renal failure in the rat. Ren Fail.

[8] Liang Y (2014). Isoflurane preconditioning ameliorates renal ischemia-reperfusion injury through antiinflammatory and antiapoptotic actions in rats. Biol Pharm Bull.

[9] Muntean DM (2013). Volatile anaesthetics and cardioprotection: lessons from animal studies. Fundam Clin Pharmacol.

[10] Redel A (2009). Comparison of isoflurane-, sevoflurane-, and desflurane-induced pre- and postconditioning against myocardial infarction in mice in vivo. Exp Biol Med (Maywood).

[11] Davis RF (1983). The effect of halothane anesthesia on myocardial necrosis, hemodynamic performance, and regional myocardial blood flow in dogs following coronary artery occlusion. Anesthesiology.

[12] Zaugg M (2014). Anesthetic cardioprotection in clinical practice from proof-of-concept to clinical applications. Curr Pharm Des.

[13] Zaugg M (2002). Differential effects of anesthetics on mitochondrial K(ATP) channel activity and cardiomyocyte protection. Anesthesiology.

[14] Mullenheim J (2001). Ketamine, but not S(+)-ketamine, blocks ischemic preconditioning in rabbit hearts in vivo. Anesthesiology.

[15] Cope DK (1997). Volatile anesthetics protect the ischemic rabbit myocardium from infarction. Anesthesiology.

[16] Kohro S (2001). Anesthetic effects on mitochondrial ATP-sensitive K channel. Anesthesiology.

[17] Wever KE (2012). Ischemic preconditioning in the animal kidney, a systematic review and meta-analysis. PLoS One.

[18] Hausenloy DJ (2007). Effect of remote ischaemic preconditioning on myocardial injury in patients undergoing coronary artery bypass graft surgery: a randomised controlled trial. Lancet.

[19] Walsh SR (2008). Ischaemic preconditioning during cardiac surgery: systematic review and meta-analysis of perioperative outcomes in randomised clinical trials. Eur J Cardiothorac Surg.

[20] Meng R (2012). Upper limb ischemic preconditioning prevents recurrent stroke in intracranial arterial stenosis. Neurology.

[21] Jones H (2014). Seven-day remote ischemic preconditioning improves local and systemic endothelial function and microcirculation in healthy humans. Am J Hypertens.

[22] Jablonski P (1983). An experimental model for assessment of renal recovery from warm ischemia. Transplantation.

[23] Wever KE (2012). Local and remote ischemic postconditionings have synergistic protective effects on renal ischemia-reperfusion injury. Transplantation.

[24] Wever KE (2011). Remote ischaemic preconditioning by brief hind limb ischaemia protects against renal ischaemia-reperfusion injury: the role of adenosine. Nephrol Dial Transplant.

[25] Burne MJ (2000). Genetic susceptibility to renal ischemia reperfusion injury revealed in a murine model. Transplantation.

[26] Guo Y (2012). Genetic background, gender, age, body temperature, and arterial blood pH have a major impact on myocardial infarct size in the mouse and need to be carefully measured and/or taken into account: results of a comprehensive analysis of determinants of infarct size in 1,074 mice. Basic Res Cardiol.

[27] Ali ZA (2007). Remote ischemic preconditioning reduces myocardial and renal injury after elective abdominal aortic aneurysm repair: a randomized controlled trial. Circulation.

[28] Wei M (2011). Repeated remote ischemic postconditioning protects against adverse left ventricular remodeling and improves survival in a rat model of myocardial infarction. Circ Res.

[29] Rohailla S (2014). Acute, delayed and chronic remote ischemic conditioning is associated with downregulation of mTOR and enhanced autophagy signaling. PLoS One.

[30] Liang Y (2015). Long-term, regular remote ischemic preconditioning improves endothelial function in patients with coronary heart disease. Braz J Med Biol Res.

